# HIV Knowledge, Attitudes, and Practices of Young People in Iran: Findings of a National Population-Based Survey in 2013

**DOI:** 10.1371/journal.pone.0161849

**Published:** 2016-09-14

**Authors:** Mostafa Shokoohi, Mohammad Karamouzian, Ali Mirzazadeh, AliAkbar Haghdoost, Ali-Ahmad Rafierad, Abbas Sedaghat, Hamid Sharifi

**Affiliations:** 1 HIV/STI Surveillance Research Center, and WHO Collaborating Center for HIV Surveillance, Institute for Futures Studies in Health, Kerman University of Medical Sciences, Kerman, Iran; 2 Epidemiology & Biostatistics, Schulich School of Medicine & Dentistry, The University of Western Ontario, London, ON, Canada; 3 School of Population and Public Health, Faculty of Medicine, University of British Columbia, Vancouver, BC, Canada; 4 Department of Epidemiology and Biostatistics, University of California San Francisco, San Francisco, CA, United States of America; 5 Modeling in Health Research Center, Institute for Futures Studies in Health, Kerman University of Medical Sciences, Kerman, Iran; 6 Center for Disease Control (CDC), Ministry of Health and Medical Education, Tehran, Iran; University of California San Francisco, UNITED STATES

## Abstract

**Introduction:**

The evidence is mixed on the HIV knowledge, attitude, and practices of youth in Iran. The aim of the current study was to assess knowledge, attitudes, and practices of Iranian youth towards HIV through a national survey.

**Materials and Methods:**

Through a cross-sectional study with multistage cluster sampling, we administered a pilot-tested standard questionnaire to assess the levels of HIV knowledge, attitudes and practices of individuals aged 15–29 years old. Participants were recruited from 13 provinces in Iran and consisted of 2456 men and 2412 women.

**Results:**

Only 37.3% of the participants had a high knowledge score. Most participants knew the main routes of HIV transmission; however, misconceptions existed about the transmission of HIV through mosquito bites across all age groups (31.7% correct response). Positive levels of attitude wereobserved among 20.7% of the participants. Most participants believed that people living with HIV (PLHIV) should be supported (88.3%) while only 46.3% were ready to share a table with them. Among those aged 19–29 years old, the main source of HIV information was mass media (69.1%), only 13.1% had ever tested for HIV, around 20.8% had ever had extramarital sex (31.7% male vs. 9.6% female),1.8% ever injected drugs (2.9% male vs. and 0.7% female). Among sexually active subjects in this age group, only 21.8% (26.1% male vs. 7.1% female) were consistent condom users.

**Conclusions:**

The findings showed that Iranian youth and young adults have relatively insufficient overall knowledge and negative attitudes about HIV and PLHIV. Novel strategies involving schools and youth’s networks could be employed to deliver a culturally sensitive sexual health program.

## Introduction

While the Middle East and North Africa (MENA) region has the lowest number of people living with HIV (PLHIV), the epidemic is expanding at a rapidly increasing rate. In the case of Iran, the estimated prevalence in the general population remains low (i.e., 0.14%), and the concentrated HIV epidemic continues to be mainly driven by injection drug use with a prevalence of 13.8% among people who inject drugs (PWID) [1, 2]. Nevertheless, the modes of transmission for HIV in Iran are changing, and rates of infection through unsafe sexual practices are increasing [2, 3].

Youth are at an increased risk of HIV and account for about half of the new HIV infections in many nations [4, 5]. Being an important period for social development, the adolescent and young adulthood stages are critical for promoting healthy attitudes and behaviors to protect young people from HIV. Their elevated risk of HIV infection has been attributed to their lack of knowledge and engagement in risky sexual and injection behaviors; calling for targeted educational interventions in improving their HIV knowledge and decreasing their risky behaviors [6]. Increasing HIV knowledge has been suggested as an effective HIV preventive behavioral intervention across different contexts. Elevating HIV knowledge creates motivation for risk reduction and has been associated with increased safe sex practices and HIV testing and treatment uptake [7].

The evidence is mixed on the HIV knowledge, attitude, and practice (KAP) of Iranian general population. A systematic review of HIV KAP studies across different sub-populations in 2011, reports relatively high scores of knowledge and attitude among various populations [8]. However, most studies in that review were heterogeneous and came from studies with small sample sizes with limited generalizability to the general Iranian population. Indeed, most previous HIV KAP studies among Iranian youth aged 15–29 that indicate a high level of knowledge and positive attitude towards HIV, are mainly limited to high school [9–12] and medical students [13, 14] in certain provinces. On the other hand, a considerable number of studies on HIV KAP among some demographics of the general and young population, suggest fairly low scores [15–18]. Therefore, given that data on youth’s KAP towards HIV play an important role indestigmatizing HIV and reducing their risky behaviors that can endure into adulthood [19] and the controversial estimates across different studies, it is critical to inform health policy makers and HIV prevention programs with reliable estimates. Therefore, through a population-based large national survey, this study tries to examine Iranian youth’s KAP towards HIV. Findings of this study have important implications for future HIV preventive efforts and policies targeting Iranian youth.

## Materials and Methods

### Study population

This cross-sectional survey was conducted in 13 (out of 31) provinces in Iran between January and March 2013. In a multistage sampling scheme, 4950 young men and women aged 15–29 years old were recruited proportionate to population size of provinces, meaning that larger provinces had larger samples.

### Sampling and data collection

Based on the Statistical Center of Iran’s provincial literacy rates [20], all 31 provinces were categorized into three strata of literacy level: low, moderate, and high. Four provinces from low literacy level, three provinces from the moderate level, and six provinces from the high literacy level were selected. Within each stratum, provinces were considered as clusters. The province of Tehran was treated as three clusters, due to its large population, and other provinces were considered as a single cluster. Therefore, considering the geographic distribution of the provinces, a total of 13 provinces (15 clusters) were purposefully selected for data collection. Assuming that 65% of the general population had a good level of HIV knowledge [8], the power of 80%, and a design effect of 5 (due to the convenient nature of the sample), and a precision level of 0.03, a sample size of 4855 was calculated. Allowing for a non-response rate of 10% (4855/(1–0.10) = 5395) andinformed by expert opinions within Ministry of Health, the calculated sample size was adjusted to 4950.

In each cluster, 330 participants were selected. Based on the population distributions reported in the 2011 census [20], 70% of the participants were recruited from the capital and 30% from the non-capital cities within province. Furthermore, within each city, 70% and 30% of the participants were selected from the urban and rural regions, respectively. Urban regions were divided into five areas (i.e., north, east, west, south, and central) and in each area, two public places were randomly chosen. Rural areas were divided into three strata based on development indicators, and one village was randomly selected from each stratum. Using quota sampling, trained interviewers selected participants from different crowded streets and public places at differenttimes of the day. This was guided by our previous research that suggested people are more likely to disclose sensitive information (e.g., sexual health-related topics) in street-based surveys compared to household- or telephone-based surveys [21].

### Survey instrument

The questionnaire was developed through a review of the HIV/AIDS literature [22, 23] and focus group discussions with HIV experts and key informants at the Ministry of Health. The questionnaire consisted of 14 questions/statements on knowledge of HIV modes of transmission, 10 questions on knowledge of HIV diagnosis, prevention, and treatment, as well as 12 questions/statements on attitudes towards HIV/AIDS. We also asked participates about high-risk sexual and drug use behaviors and their sources of HIV/AIDS knowledge. The last section of the questionnaire collected data on participants’ socio-demographic background.

The questionnaire was pilot-tested with 150 participants in three provinces across different strata to ensure clarity, relevance, and accessibility. The content validity was assessed by expert opinion, and the internal reliability was assessed by measuring the Cronbach alpha coefficient; 0.781 for knowledge of HIV modes of transmission, 0.751 for knowledge of HIV diagnosis, prevention, and treatment, and 0.867 for attitudes towards HIV/AIDS. Knowledge questions were scored by “yes”, “no”, and “don’t know”. Overall knowledge was determined by aggregating correct answers from all questions (0–24 for questions on HIV/AIDS knowledge). Based on previous research in this field [6], three levels including (I) low (≤12), (II) medium (13–18), and (III) high (19–24) were specified to assess participants’HIV/AIDS knowledge. Attitudes statements were scored by “agreed”, “disagreed”, and “no idea”. Overall attitudes were determined by aggregating all positive responses ranging from 0 to 12. Levels of (I) negative (0–6 positive responses), (II) neutral (7–9 positive responses) and (III) positive (10–12 positive responses) were quantified.

### Statistical analysis

Data were entered via double data entry into Stata v.11 software (StataCorp; College Station, Texas, USA), cleaned, and checked for errors before coding. As participants were recruited from different regions, clustering effects were adjusted using Stata survey package. Data were presented as relative frequencies and 95% confidence intervals (CI) for categorical variables and means with 95% CI for quantitative variables. Participants’ knowledge, attitude, and behaviors in different age groups were compared using chi-square tests. All P-values were two-sided and values less than 5% were considered as statistically significant.

### Ethical considerations

Verbal informed consent forms were obtained from all eligible subjects. Given the sensitivity of the topic and the street-based nature of the sample, acquiring written informed consent was not feasible. However, the interviewers explained the informed consent form to the participants and signed the data collection forms to confirm that they had obtained verbal informed consent for all participants. Participants were also briefed about the objectives of the survey and the anonymous nature of data. For minor participants, verbal informed consent was obtained from their caretaker. The ethics committee of the Kerman University of Medical Sciences approved the study protocol and waived the need for written informed consent (Reference Number: K/93/205).

## Results

### Demographic information

A total of 4950 (2456 men and 2412 women) individuals were approached, out of which 4868 participants completed the survey. The mean (standard deviation (SD)) age was 21.8 (5.6) years; 33.4% (n = 1622) were 15–18 years old, 35.6% (n = 1733) were 19–24 years old, and 31% (n = 1507) were 25–29 years old. Most participants were single (70.7%), had some levels of university educations (58.4%), and around 7.3% reported to be unemployed (Table 1).

**Table 1:** Socio-demographic characteristics of the study population (N = 4868).

Variable	n (%)
**Age**	
15–18	1622 (33.4)
19–24	1733 (35.6)
25–29	1507 (31.0)
Mean (±SD)*	21.8 (±5.6)
**Sex**	
Male	2456 (50.5)
Female	2412 (49.6)
**Marital status**	
Single	3407 (70.7)
Married	1350 (28.0)
Widowed/Divorced	63 (1.3)
**Education**	
Primary or less	1573 (32.5)
Secondary	440 (9.1)
University student or graduate	2824 (58.4)
**Employment Status**	
Pupil	1356 (28.0)
University student	838 (17.3)
Self-employed	744 (15.4)
Housekeeper	640 (13.2)
Government employee	371 (7.7)
Unemployed	355 (7.3)
Laborer	332 (6.9)
Other	204 (4.2)

*SD: Standard Deviation.

### HIV/AIDS knowledge

HIV knowledge was assessed in two parts and participants’ responses are presented in Table 2.

**Table 2:** Knowledge of HIV/AIDS transmission and prevention (N = 4868).

Statements (correct response)	15–18 years	19–24 years	25–29 years	Total	P-value
Knowledge of modes of transmission*					
Sharing food and water utensils with PLHIV^a^	1028 (63.8)	1212 (70.5)	1141 (76.4)	3381 (70.1)	<0.001
HIV-infected pregnant woman to her baby (i.e., pregnancy)^b^	1246 (77.0)	1365 (79.6)	1233 (83.1)	3844 (79.8)	0.006
HIV-infected pregnant woman to her baby (i.e., breastfeeding)^b^	884 (55.0)	876 (51.8)	694 (47.4)	2454 (51.5)	0.014
Using the personal belongings and toiletries of PLHIV^a^	1108 (69.0)	1251 (73.5)	1123 (76.6)	3482 (72.9)	0.005
Using the haircut kit of PLHIV^a^	1208 (75.1)	1437 (84.2)	1297 (88.4)	3942 (82.4)	<0.001
Piercing with HIV-infected equipment^b^	1277 (79.1)	1454 (85.0)	1328 (90.3)	4059 (84.6)	<0.001
Using dental instruments used for PLHIV^b^	1119 (69.9)	1314 (77.1)	1253 (85.1)	3686 (77.1)	<0.001
Using public bathrooms^a^	974 (60.8)	1137 (66.4)	1046 (70.7)	3157 (65.9)	<0.001
Using unsterile equipment of tattoo and bloodletting^b^	1242 (78.3)	1417 (83.8)	1307 (88.6)	3966 (83.4)	<0.001
Mosquito/insect bite^a^	542 (33.9)	531 (31.2)	442 (29.9)	1515 (31.7)	0.121
Kissing and/or hugging PLHIV^a^	1035 (64.5)	1226 (71.9)	1106 (74.7)	3367 (70.3)	<0.001
Sharing injection needles/syringes with PLHIV^b^	1377 (85.4)	1515 (88.3)	1359 (91.1)	4251 (88.2)	0.003
Contacting sneeze, cough or saliva of PLHIV^a^	764 (47.5)	926 (54.1)	854 (57.5)	2544 (52.9)	<0.001
Unprotected sex (i.e., without condom) with PLHIV^b^	1334 (82.5)	1545 (89.4)	1380 (91.9)	4259 (87.9)	<0.001
**Knowledge of HIV prevention, diagnosis, and treatment**					
One can identify PLHIV, by their physical appearance^a^	1167 (72.0)	1396 (80.7)	1249 (83.2)	3812 (78.6)	<0.001
Treatment can reduce the chance of HIV transmission^b^	585 (36.4)	589 (34.3)	526 (35.3)	1700 (35.3)	0.611
Early diagnosis/treatment increases PLHIV’s life expectancy^b^	972 (60.6)	1168 (68.0)	1128 (75.7)	3268 (67.9)	<0.001
Blood testing is the only definite diagnosis of HIV infection^b^	1102 (68.4)	1262 (73.2)	1185 (79.4)	3549 (73.5)	<0.001
There exists a cure for AIDS^a^	1104 (68.6)	1185 (69.2)	1107 (74.4)	3396 (70.6)	0.010
Using condom reduces the chance of HIV transmission^b^	1038 (64.4)	1216 (71.0)	1167 (78.2)	3421 (71.0)	<0.001
PLHIV can have a normal life by following a healthy diet and life style, and taking their treatments^b^	753 (46.8)	947 (55.2)	955 (64.1)	2655 (55.2)	<0.001
There exists a vaccine that prevents AIDS^a^	777 (48.4)	929 (54.0)	941 (63.1)	2647 (55.0)	<0.001
Having multiple sex partners increases the chance of HIV infection^b^	1118 (69.2)	1290 (75.0)	1191 (79.3)	3599 (74.4)	<0.001
Presence of an STD (e.g., genital ulcers) increases the chance of HIV infection^b^	1016 (62.8)	1229 (71.3)	1142 (75.9)	3387 (69.9)	<0.001

*PLHIV: people living with HIV; STD: sexually transmitted diseases; Data are n (%)

^a^For these questions, “No” is the correct answer

^b^For these questions, “Yes” is the correct answer.

#### Part I: Knowledge of HIV modes of transmission

Participants aged 25–29 years were significantly more knowledgeable about HIV modes of transmission compared to other age groups. Most participants knew the main routes of HIV transmission and could correctly identify the risk of piercing (84.6%) and tattoo (83.4%) with HIV-infected equipment, sharing injection needles/syringes (88.2%), and unprotected sex with PLHIV (87.9%) in transmitting HIV. However, only around half of the participants knew that HIV can be transmitted through breastfeeding (51.5%), or that it cannot be spread through the sneeze, cough or saliva of PLHIV (52.9%). Moreover, misconceptions existed about the transmission of HIV through mosquito/insect bites across all age groups (31.7% correct response).

#### Part II: Knowledge of HIV prevention, diagnosis, and treatment

Most participants knew that PLHIV cannot be identified by their appearance (78.6%), and HIV testing is the only diagnostic measure (73.5%). Around 70% knew that condom use reduces the chance of HIV transmission. Most participants knew that having multiple sexual partners and pre-existence of sexually transmitted diseases (STD) could increase the chance of HIV infection (74.4% and 69.9%, respectively). Participants’ knowledge about HIV treatment was relatively low, and 55.0% believed that an HIV vaccine exists. More than one-third (35.3%) of the participants knew that antiretroviral therapy could reduce the chance of HIV transmission. Around two-third (67.9%) of them knew that early diagnosis and treatment increases the life expectancy of PLHIV (Table 2).

### Attitudes towards HIV/AIDS

Overall, only 38.4% declared tolerance for working or studying with PLHIV. More than half of the participants rejected the misconceptions of viewing HIV as a fair punishment for the sins of the past (57.0%) or quarantining PLHIV as the best HIV preventative intervention (61.8%). Most participants believed that PLHIV should be supported and receive treatments (88.3%), and 47.7% revoked feelings of disgust when thinking about kissing or hugging PLHIV. Around two-third (61.7%) of the participants declared that AIDS is not only exclusive to high-risk populations such as PWID or female sex workers, and 51.4% would not break up their contacts with PLHIV. The poorest attitudes were observed towards sharing a table with PLHIV (46.3% positive attitudes) and feelings of despair in the case of testing positive for HIV (40.1% positive attitudes) (Table 3).

**Table 3:** Attitudes towards people living with HIV (n = 4868).

Statements (response for positive attitude)*	15–18 years	19–24 years	25–29 years	Total	P-value
**Attitude towards PLHIV**					
I can work/study with an HIV-infected colleague/classmate^b^	550 (34.0)	712 (41.2)	603 (40.1)	1865 (38.4)	0.006
HIV is not a punishment for the sins and immoralities of the past^a^	820 (50.7)	1030 (60.0)	904 (60.4)	2754 (57.0)	0.001
Quarantine of PLHIV is not the best way to prevent HIV^a^	909 (56.3)	1142 (66.4)	934 (62.4)	2985 (61.8)	<0.001
We should not avoid/stay away from a family with an HIV-infected member^b^	1013 (62.6)	1194 (69.4)	1012 (67.8)	3219 (66.6)	0.002
PLHIV have to be supported and receive treatment^b^	1393 (86.5)	1503 (87.2)	1364 (91.4)	4260 (88.3)	0.005
PLHIV do not like to infect others^b^	953 (59.6)	976 (56.9)	838 (56.7)	2767 (57.8)	0.229
Kissing or hugging people living with HIV is not disgusting^b^	719 (44.6)	856 (50.2)	717 (48.1)	2292 (47.7)	0.005
I prefer not to break my contacts with PLHIV^b^	767 (47.6)	942 (54.9)	767 (51.5)	2476 (51.4)	0.013
PLHIV do not bring disgrace and shame of their family^b^	926 (57.6)	1104 (64.4)	896 (60.1)	2926 (60.8)	0.002
I can share a table PLHIV^b^	673 (42.1)	847 (49.3)	705 (47.3)	2225 (46.3)	0.007
AIDS is not only the problem of people who inject drugs and people with unrestrained sexual practices (e.g., female sex workers)^b^	888 (55.0)	1046 (60.8)	1045 (70.0)	2979 (61.7)	<0.001
If I become infected with HIV, my life is not over^b^	665 (41.1)	682 (39.6)	594 (39.6)	1941 (40.1)	0.667

*PLHIV: people living with HIV; Data are n (%)

^a^For these questions, “No” shows a positive attitude

^b^For these questions, “Yes” shows a positive attitude.

### Overall knowledge and attitude

**Fig 1:**
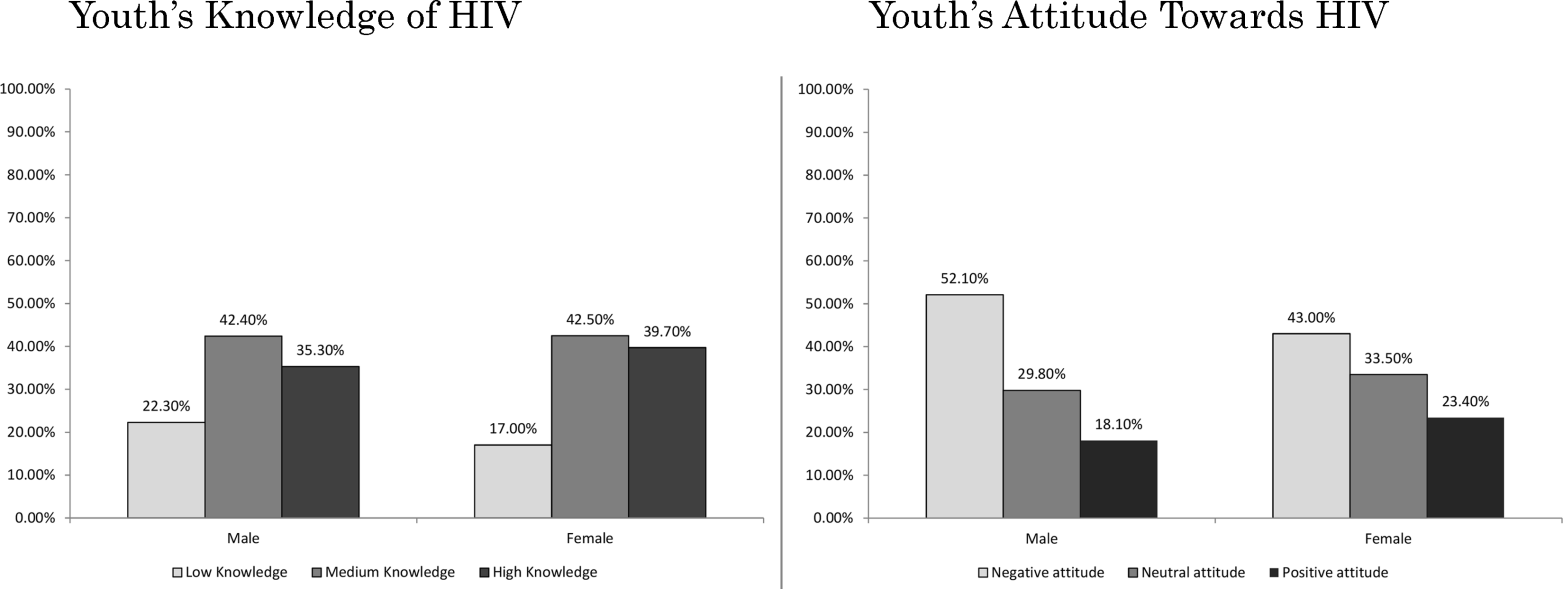
HIV-related Knowledge and attitude scores among young men and women in Iran.

Details of knowledge and attitude scores stratified by age, residence type, and type of cities are presented in Table 4. Moreover, gender differences in knowledge and attitude scores are presented in Fig 1.

**Table 4:** Overall knowledge and attitude towards HIV/AIDS.

	Knowledge				Attitude			
	Low (12 or less)	Medium (13–18)	High (19 or more)	P-value	Negative (6 or less)	Neutral (7–9)	Positive (10 or more)	P-value
Total*	958 (19.7)	2095 (43.0)	1815 (37.3)	—	2317 (47.6)	1542 (31.7)	1009 (20.7)	—
**Age groups**				<0.001				0.005
15–18	418 (25.8)	769 (47.4)	435 (26.8)		856 (52.8)	504 (31.1)	262 (16.2)	
19–24	334 (19.3)	748 (43.2)	651 (37.6)		777 (44.8)	545 (31.4)	411 (23.7)	
25–29	205 (13.6)	577 (38.3)	725 (48.1)		682 (45.3)	490 (32.5)	335 (22.2)	
**Residence type**				<0.001				<0.001
Rural	338 (24.6)	609 (44.4)	426 (31.0)		751 (54.7)	377 (27.5)	245 (17.8)	
Urban	620 (17.7)	1486 (42.5)	1389 (39.7)		1566 (44.8)	1165 (33.3)	764 (21.9)	
**Type of cities**				0.745				0.051
Capital cities	592 (20.1)	1259 (42.9)	1087 (37.0)		1457 (49.6)	914 (31.1)	567 (19.3)	
Non-capital cities	366 (19.0)	836 (43.3)	728 (37.7)		860 (44.6)	628 (32.5)	442 (22.9)	

*Data are n (%).

A total of 19.7%, 43.0%, and 37.3% of the participants had low, medium, and high levels of HIV knowledge, respectively. Older (48.1% vs. 26.8%; P-value<0.001), female (39.1% vs. 35.3%; P-value = 0.006), and urban (39.7% vs. 31.0%; P-value<0.001) participants were significantly more knowledgeable about HIV. Moreover, 47.6%, 31.7%, and 20.7% of the participants had negative, neutral, and positive attitudes towards PLHIV, respectively. Attitudes were also significantly better among older (23.7% vs. 16.2%; P-value<0.005), female (23.4% vs. 18.1%; P-value<0.001), and urban (21.9% vs. 17.8%; P-value<0.001) participants.

### HIV/AIDS risk behaviors

Data on HIV-related risk behaviors was only collected for subjects aged 19–29 years. Ever drug injection was reported by 1.8% (95% CI: 1.2, 2.7) of participants (2.9% male vs. 0.7% female) and ever needle/syringe sharing was reported by 0.4% (95% CI: 0.2, 1.0) of the participants. Extramarital sex or temporary marriage was reported by 20.8% (95% CI: 16.3, 26.2) of the youth (31.7% male vs. 9.6% female) and mean age at the first extramarital sex among sexually active participants was 19.0 (95% CI: 18.5, 19.5) years. Only 21.8% were consistent condom users (26.1% male vs. 7.1% female) and 27.6% had never or rarely used condoms (24.4% male vs. 38.3% female). While younger participants reported lower condom use practices, there was no significant difference between the two age groups (P-value = 0.177). Main reasons for non-condom use were inaccessibility (38.4%), unnecessity (32.3%), and disinterest (28.8%). Around one-third of the participants (35.4%; 95% CI: 30.1, 41.1) reported condom use in their last sexual encounter (38.2% male vs. 25.9% female). About 40% had ever used alcohol or drugs during or before sex (39.8% male vs. 40.3% female). A total of 86.9% (95% CI: 84.4, 89.1) had never tested for HIV, and only 6.5% (8.1% male vs. 4.9% female) had tested for HIV during the previous year. Media (69.1%), acquaintances (40.9%), and the web (39.9%) were reported as principal sources of obtaining information about HIV (Table 5).

**Table 5:** HIV/AIDS risky behavior practices in a survey among young people in Iran.

Variables (number of valid responses)	19–24 years	25–29 years	Total	P-value^Ɨ^
**Ever injected illicit or stimulants drugs** (n = 3000)	1.9 (1.2, 3.0)*	1.7 (1.0, 2.9)	1.8 (1.2, 2.7)	0.721
**Male** (n = 1491)	2.9 (1.9, 4.6)	2.8 (1.6, 5.0)	2.9 (1.9, 4.3)	0.895
**Female** (n = 1504)	0.9 (0.3, 2.5)	0.6 (0.2, 2.0)	0.7 (0.3, 1.8)	0.536
**Ever used shared/unsterile syringes/needles** (n = 3240)	0.5 (0.2, 1.1)	0.3 (0.1, 1.3)	0.4 (0.2, 1.0)	0.495
**Male** (n = 1628)	0.8 (0.3, 1.9)	0.7 (0.2, 2.6)	0.7 (0.3, 1.9)	0.658
**Female** (n = 1612)	0.1 (0.0, 1.0)	0.0	0.1 (0.0, 0.5)	0.371
**Extramarital sex or temporary marriage** (n = 3040)	19.8 (15.3,25.1)	22.0 (16.7,28.3)	20.8 (16.3,26.2)	0.214
**Male** (n = 1537)	30.0 (23.2,37.8)	33.5 (25.0,43.2)	31.7 (24.7,39.6)	0.291
**Female** (n = 1503)	9.5 (6.6,13.7)	9.8 (6.4,14.7)	9.6 (6.7,13.7)	0.872
**Mean age at the first extramarital sex** (n = 413)	18.4 (17.8, 18.9)	20.4 (19.6, 21.1)	19.3 (18.8, 19.8)	<0.001
**Male** (n = 303)	18.0 (17.3, 18.7)	20.2 (19.5, 20.9)	19.0 (18.5, 19.5)	<0.001
**Female** (n = 109)	19.5 (18.6, 20.5)	20.9 (19.8, 22.0)	20.2 (19.6, 20.8)	0.022
**Using condom during sex** (n = 616)				0.177
Always	19.0 (14.1,25.1)	24.5 (19.5,30.4)	21.8 (17.4,26.8)	
Often	14.2 (9.3,21.1)	16.7 (11.2,24.2)	15.4 (11.3,20.8)	
Sometimes	35.8 (29.5,42.6)	34.6 (27.9,42.1)	35.2 (29.9,41.0)	
Rarely/Never	31.0 (26.6,35.7)	24.2 (20.1,28.8)	27.6 (24.2,31.3)	
**Male** (n = 475)				0.351
Always	23.0 (16.8,30.6)	29.2 (23.1,36.1)	26.1 (20.8,32.2)	
Often	16.2 (10.7,23.7)	15.8 (10.2,23.8)	16.0 (11.3,22.2)	
Sometimes	34.0 (26.9,42.0)	32.9 (25.5,41.3)	33.5 (26.9,40.7)	
Rarely/Never	26.8 (21.8,32.5)	22.1 (17.4,27.6)	24.4 (20.9,28.3)	
**Female** (n = 141)				
Always	6.7 (2.0,20.3)	7.6 (3.5,15.8)	7.1 (3.4,14.4)	0.189
Often	8.0 (3.5,17.1)	19.7 (9.6,36.1)	13.5 (7.4,23.3)	
Sometimes	41.3 (28.4,55.6)	40.9 (27.2,56.2)	41.1 (30.6,52.6)	
Rarely/Never	44.0 (32.9,55.8)	31.8 (23.8,41.1)	38.3 (31.1,46.0)	
**Reasons for inconsistent condom use** (n = 632)				
It was not accessible	40.6 (35.6,45.8)	36.3 (30.6,42.5)	38.4 (33.8,43.3)	0.134
It is too expensive	10.1 (6.1,16.0)	4.1 (2.2,7.6)	7.1 (4.3,11.6)	<0.001
My partner objected	20.8 (17.4,24.6)	9.2 (4.8,16.9)	15.0 (10.9,20.4)	0.024
I do not like it	28.3 (21.8,35.9)	29.3 (23.9,35.4)	28.8 (23.8,34.4)	0.773
I used something else	4.4 (2.9,6.6)	4.5 (2.6,7.5)	4.4 (3.3,5.9)	0.971
I did not think it was necessary	32.4 (25.4,40.3)	32.2 (26.4,38.6)	32.3 (26.7,38.4)	0.947
**Using condom in the last sexual contact** (n = 616)				0.090
Yes	31.8 (25.0,39.5)	39.0 (33.7,44.5)	35.4 (30.1,41.1)	
No	59.1 (53.9,64.1)	48.4 (42.8,54.0)	53.7 (48.9,58.5)	
I do not remember	9.1 (5.6,14.5)	12.7 (8.7,18.0)	10.9 (8.5,13.8)	
**Male** (n = 477)				0.277
Yes	35.0 (26.9,44.2)	41.2 (34.4,48.2)	38.2 (32.5,44.2)	
No	55.6 (49.5,61.4)	46.5 (39.9,53.2)	50.9 (45.5,56.3)	
I do not remember	9.4 (5.2,16.4)	12.3 (8.3,18.0)	10.9 (8.5,13.8)	
**Female** (n = 139)				0.321
Yes	21.6 (14.1,31.7)	30.8 (19.0,45.8)	25.9 (19.8,33.1)	
No	70.3 (57.6,80.5)	55.4 (38.8,70.8)	63.3 (51.3,73.8)	
I do not remember	8.1 (1.9,28.3)	13.8 (6.0,28.7)	10.8 (4.9,22.3)	
**Ever used alcohol or drugs during/before sex** (n = 614)				0.620
Yes	40.8 (35.6,46.3)	39.0 (31.7,46.8)	39.9 (34.6,45.4)	
No	53.9 (47.9,59.8)	54.5 (47.3,61.6)	54.2 (48.6,59.7)	
I do not remember	5.2 (2.8,9.4)	6.5 (4.1,10.1)	5.9 (3.7,9.3)	
**Male** (n = 475)				
Yes	42.2 (35.2,49.6)	37.4 (29.6,46.0)	39.8 (33.4,46.5)	0.210
No	53.0 (45.7,60.2)	55.6 (48.7,62.2)	54.3 (48.2,60.3)	
I do not remember	4.7 (2.7,8.2)	7.0 (3.8,12.4)	5.9 (3.5,9.6)	
**Female** (n = 139)				
Yes	36.5 (22.3,53.5)	44.6 (28.3,62.1)	40.3 (28.1,53.9)	0.712
No	56.8 (41.5,70.9)	50.8 (32.3,69.0)	54.0 (40.4,67.0)	
I do not remember	6.8 (2.3,18.2)	4.6 (0.9,19.9)	5.8 (2.5,12.9)	
**HIV testing** (n = 3074)				<0.001
Never tested	89.9 (87.7,91.7)	83.4 (79.4,86.8)	86.9 (84.4,89.1)	
Tested in the past 12 months	5.2 (4.1,6.5)	8.0 (6.4,9.9)	6.5 (5.5,7.6)	
Tested before the past 12 month	4.9 (3.5,6.8)	8.6 (6.2,11.8)	6.6 (5.0,8.7)	
**Male** (n = 1549)				
Never tested	88.0 (84.8,90.6)	80.1 (74.6,84.6)	84.2 (81.0,87.1)	0.003
Tested in the past 12 months	6.1 (4.3,8.6)	10.2 (7.3,14.1)	8.1 (6.2,10.5)	
Tested before the past 12 month	5.9 (4.4,7.8)	9.7 (6.8,13.6)	7.7 (6.0,9.9)	
**Female** (n = 1525)				
Never tested	91.8 (89.7,93.4)	87.0 (81.9,90.8)	89.6 (86.5,92.0)	0.008
Tested in the past 12 months	4.2 (3.3,5.4)	5.6 (4.0,7.8)	4.9 (3.9,6.1)	
Tested before the past 12 month	4.0 (2.5,6.4)	7.4 (4.8,11.4)	5.6 (3.8,8.2)	
**Source of obtaining information about HIV** (n = 3240)				
Family, friends and acquaintances	40.8 (36.5,45.2)	41.1 (36.2,46.1)	40.9 (36.6,45.4)	0.831
Media (e.g., TV, radio, newspaper)	65.8 (61.4,70.1)	72.9 (67.0,78.2)	69.1 (64.6,73.4)	0.012
Internet	42.2 (37.9,46.6)	37.4 (33.3,41.6)	39.9 (36.3,43.7)	0.037
Health centers and hospitals	31.3 (27.6,35.3)	36.5 (32.8,40.4)	33.7 (30.6,37.0)	0.018
Schools or universities	40.6 (37.1,44.3)	29.5 (26.5,32.6)	35.4 (32.5,38.5)	<0.001
Do not know much about HIV	11.0 (8.8,13.7)	9.8 (7.5,12.8)	10.5 (8.6,12.6)	0.436
Other	1.6 (0.9,2.7)	1.7 (1.1,2.6)	1.6 (1.1,2.3)	0.843

*Data are % (95% CI)

Ɨ P-values were obtained from Chi-Square test.

## Discussion

This study revealed that a considerable subgroup of Iranian youth know little about HIV. While most identified the basic routes of transmission, approximately half of the participants did not know that HIV cannot be transmitted through sneezes of PLHIV, and a large proportion of them falsely indicated that mosquito bites can transmit the HIV. Moreover, youth’s knowledge of HIV treatment was similarly low as most did not know that HIV treatment reduces the chances of transmission and around 55% believed that an HIV vaccine exists. Our findings indicate a lower knowledge score compared to most previous studies in Iran. A systematic review on the knowledge and attitude of Iranians (2011) towards HIV, reported a mean knowledge score of 67.5, and attitude score of 68 (on a scale of 100) [8]. This high level of knowledge and positive attitude, in comparison with our study, could be due to their large sample of high school and medical students, university professors, and nurses that may have a higher level of HIV knowledge and a better attitude towards PLHIV. Other population-based studies that have recruited young participants and reported higher HIV knowledge and attitude scores are also limited to western provinces [24], and convenience volunteer samples [25].

HIV knowledge and attitudes scores were higher in urban settings. This could be explained by the uneven distribution of HIV educational campaigns across different regions with urban areas receiving more attention. It could also be related to the overall higher knowledge level of urban residents. We also observed that women were significantly more knowledgeable that men and had more positive attitudes towards HIV and PLHIV. Such findings have been reported in several studies elsewhere [26–29] and been attributed to women’s higher health consciousness and levels of empathy compared to men. As expected, knowledge and positive attitudes about HIV increased with age which could be attributed to older people’s larger interest in seeking sexual health information or higher exposure to sexual health education (e.g., premarital mandatory trainings on sexual health and HIV).

Participants’ limited knowledge about HIV could be attributed to their lack of access to sexual health information. Media was reported as our participants’ main source of seeking HIV-related sexual health information. However, similar to other conservative contexts where open discussions of sexually-related topics are challenging and sensitive [6], Iranian media has not taken a proactive role in educating the public about HIV and other sexually transmitted infections (STI). While a recent increase in the number of HIV-related documentaries (e.g., Shock, Red Ribbon) is promising and the media has started conversations on destigmatizing HIV-related topics, there is much room for improvement. Similar to other studies [30], family members were reported as the second most significant source of information on HIV-related topics. Worldwide, youth are often viewed as immature individuals who are not ‘ready’ to make the ‘right’ decisions regarding their sexual lives, and parents are expected to monitor and inform their decision about sexual and reproductive health [31]. However, in the context of Iran and several other conservative settings, parents often try to stick to the socio-cultural norms around sexuality (e.g., abstinence, delaying first sexual experience) and discussions around such topics remain very limited if they ever take place [31, 32]. Moreover, many parents are often not equipped with sufficient knowledge to provide their children with sexual health education in the context of HIV [33]. Additionally, only one-third of youth reported school as a source of information on HIV, which points to youth’s limited exposure to sexual health education in school settings. Indeed, Iranian youth’s primary official exposure to sexual health information including HIV areas, has been either through very limited topics in school curricula, pre-marital mandatory courses, a one-credit undergraduate level course on family planning—which was recently suspended—or the various interventions of the HIV/STI office at the Ministry of Health (MOH).

Unfortunately, youth’s low knowledge of HIV had translated into negative attitudes whereas only one-fifth of the participants had a positive attitude towards PLHIV. Studies in Iran have indicated how insufficient knowledge of HIV transmission and prevention leads to less positive attitudes towards HIV and PLHIV [16]. It seems that while youth were empathetic towards PLHIV, they still preferred to avoid close contact with them. This could be illustrated by participants’ favorable attitudes on the rights of PLHIV to access treatment or disapproval of shunning behaviors towards PLHIV and their families, but not wanting to kiss them or share a table or workspace with them. Such poor attitudes have been reported in similar studies in Iran [16] where out of 4641 high school students, around half disapproved of PLHIV’s presence at schools saying that they would avoid any physical contact with them. Similar negative attitudinal problems have been reported among youth from other Middle Eastern countries such as United Arab Emirates [34], where 85% of university students had a negative attitude towards PLHIV. This could be attributed to the similar conservative atmosphere of these countries regarding HIV education, and also highlights the importance of considering such attitudes in developing future HIV strategic plans.

While poor attitudes towards PLHIV can lead to PLHIV’s experiences of discrimination across various settings, positive attitudes towards PLHIV have been associated with a willingness for HIV testing [35, 36]. This was also evident in our study where poor attitudes towards PLHIV was observed next to a very low ever HIV testing prevalence. While barriers to HIV testing among Iranian youth are complicated and not yet fully understood, low uptakes of HIV testing among them could be attributed to several reasons such as confidentiality concerns, anticipated stigma, low HIV risk perceptions in youth, and clinic locations and hours of operation [37]. Such low HIV testing prevalence is very concerning as frequent HIV testing practices have been associated with a reduced risk of HIV transmission and lower risky behavior profiles among youth [16].

Moreover, despite youth’s knowledge about reduced risk of HIV transmission via safe sex practices, less than one-third of them had used a condom in their last sexual encounter, and only 21.8% were consistent condom users. Moreover, around 30% of the participants perceived consistent condom use as unnecessary. Although condoms are widely accessible in pharmacies with a relatively affordable price, accessibility was reported as the main reason for youth’s inconsistent condom use. While barriers to condom use among Iranian youth are less understood, profound levels of anticipated or enacted stigma associated with obtaining condoms among Iranian youth could create a reluctance in purchasing condoms. Such stigmas could be rooted in the religious and socio-cultural norms around sexual practices of youth—unmarried youth in particular—that do not welcome youth’s engagement in extramarital sexual practices.

Young men were much more likely to use a condom compared to young women. This calls for further gender-sensitive condom promotion interventions and could be due to young women’s lower negotiation skills or self-perceived risk of HIV compared to men [38, 39]. Moreover, around one-fifth of participants reported having extramarital or pre-marital relationships with a significantly higher prevalence among young men. Studies have shown how existing gender inequalities around youth sexuality may lead to different sexual expectations for young men and women. For example, young men may be expected to gain sexual experience by starting sexual relationships at an earlier age or having multiple sexual partners; behaviors that may be celebrated among their peers or fathers [31].

We would like to acknowledge the limitations of this study. Although a large national sample of adults was recruited through a cluster randomized sampling approach, the street-based nature of the sample may introduce bias to our findings and limit its generalizability to some extent. However, our prior research in Iran indicates that in the context of asking culturally sensitive question, more accurate responses are obtained through street-based surveys compared with household-level and telephone-based surveys [21, 40]. Moreover, we used a three-point scale to assess attitudes towards HIV and PLHIV. While five-point scales may reflect a more precise view of the participants, fearing participants’ inability to think in such wider scale, a three-point scale was used. Lastly, social desirability bias could not be ruled out due to the self-reported nature of the data. However, the anonymity of the questionnaires and using experienced interviewers may have encouraged the participants to provide honest responses. Overall, given the scope of the study, we believe our findings have important implications for both research and policy.

### Conclusions

Our study re-emphasizes the findings of the previous body of evidence suggesting that despite being a crucial foundation in addressing the epidemic, knowledge of HIV among Iranian youth is insufficient in positively influencing their attitudes and risky sexual practices. We observed a low knowledge of HIV and fairly negative attitudes towards PLHIV among our participants. Given their limited knowledge, youth with risky sexual practices at an early age are being sent off to the society without adequate and accurate knowledge of STI and sexual health. Low levels of condom use and HIV testing among our participants are also concerning and call for targeted gender-sensitive interventions to increase condom use and HIV testing availability and acceptability among young men and women. Schools and universities are of utmost importance in reaching this goal and should be able to take a more prominent and proactive role in educating youth about sexual health and HIV-related risky behaviors. Healthcare providers and teachers, in particular, should be equipped with required training and knowledge on HIV-related topics and should take on an active responsibility in providing quality sexual educations to youth. Future research and intervention on HIV health promotion should focus on the further understanding of how socio-cultural and religious value systems affect youth’s sexual lifestyle and information access. Strategic plans should also prioritize involving the key individuals in youth’s networks (e.g., parents, teachers, and peers) in HIV education programs.
